# Case Report: Metastatic colorectal cancer with ALK–CEP44 fusion and rapid resistance development

**DOI:** 10.3389/fonc.2025.1613235

**Published:** 2025-06-18

**Authors:** Silvan Hofmann, Claudine Egger, Silke Potthast, Martin Zoche, Andreas Wicki, Tarun Mehra

**Affiliations:** ^1^ Department of Internal Medicine, Section of Medical Oncology and Hematology, Spital Limmattal, Schlieren, Switzerland; ^2^ Department of Radiology, Spital Limmattal, Schlieren, Switzerland; ^3^ Department of Pathology and Molecular Pathology, University Hospital Zurich (USZ) and University of Zurich (UZH), Zurich, Switzerland; ^4^ Department of Medical Oncology and Hematology, University Hospital Zurich (USZ) and University of Zurich (UZH), Zurich, Switzerland

**Keywords:** colorectal cancer, ALK gene mutation, targeted therapy, drug resistance, case report

## Abstract

**Background:**

Colorectal cancer rarely harbors rearrangements in the *ALK* gene, and the therapeutic significance of non-canonical or functionally unclear *ALK* fusions remains poorly defined. We report a case of metastatic CRC with an ALK–CEP44 fusion not previously described in this tumor type, associated ALK overexpression, a notable clinical response to the ALK inhibitor alectinib, and rapid development of multiple ALK resistance mutations.

**Case Summary:**

A 60-year-old male patient was diagnosed with stage IIIA right-sided CRC. Six months after adjuvant chemotherapy, he developed liver metastases. Comprehensive molecular profiling revealed strong ALK expression, a novel *ALK–*CEP44 fusion predicted to lack a functional kinase domain, and additional ALK alterations. Palliative chemotherapy induced a temporary response. Upon progression, treatment with alectinib led to rapid clinical, radiological and biochemical improvement. However, disease progression recurred shortly thereafter, and next-generation sequencing revealed four resistance-associated *ALK* mutations. The patient ultimately died due to progressive liver failure.

**Conclusion:**

ALK-targeted therapy may provide benefit in selected CRC cases with atypical *ALK* alterations, even when the oncogenic role is uncertain. Comprehensive molecular profiling and timely therapeutic decisions are essential in managing such rare and complex cases.

## Introduction

The treatment of metastatic colorectal cancer has seen some limited advances with targeted agents (1). Most druggable driver mutations are rare in colorectal cancer, with exception of KRAS mutations, occurring in around 50% of mCRC. However, the benefit of KRAS targeted agents has remained marginal so far (1, 2). Therefore, it is essential to search for and address the occasional druggable driver mutation which may provide a relevant benefit to selected patients. Certain molecular subtypes, including rare anaplastic lymphoma kinase (*ALK*) gene rearrangements, remain poorly characterized and require individualized treatment approaches. *ALK* fusions are most frequently associated with non-small cell lung cancer (NSCLC) but have also been identified in various other tumor types, including gastrointestinal cancers (3). The incidence of *ALK* gene alterations in CRC is estimated at less than 1% (4). They are typically detected only when an extended next-generation-sequencing (NGS) panel is performed, as routine screening for these alterations remains uncommon.

*ALK*-positive CRC is frequently associated with right-sided tumors, high microsatellite instability (MSI-H), and KRAS/BRAF wild-type status (3, 5). Notably, *ALK*-positive CRC may exhibit distinct clinical behaviors, including a more aggressive course and variable responses to standard chemotherapy (6). Case reports and small case series suggest that patients with *ALK*-positive mCRC may benefit significantly from *ALK*-targeted therapies, with partial responses observed in a few cases (5–9). These findings highlight the importance of comprehensive molecular profiling to guide therapeutic decisions in this rare subset of CRC.

This report presents a unique case of mCRC with ALK expression by immunohistochemistry (IHC), multiple ALK mutations and a novel fusion involving *ALK* and the centrosomal protein 44 (CEP44), which structural analysis suggests being non-functional. Despite this complex and ultimately difficult to interpret genotype, the patient exhibited a significant response to alectinib, highlighting the complexity of *ALK*-driven tumor biology and the difficulty of predicting treatment response in CRC. The case underscores the challenges of managing rare *ALK*-positive CRC, including the rapid development of resistance mutations following targeted therapy, and emphasizes the need for further research on response prediction and therapeutic strategies addressing ALK alterations.

## Case presentation

A 60-year-old male patient presented in March 2023 for a screening colonoscopy. During the procedure, an adenocarcinoma of the distal ascending colon was identified. The patient was in good health and asymptomatic at the time. In April 2023, a laparoscopic right hemicolectomy was performed, with an uneventful recovery. Histopathological evaluation confirmed a poorly differentiated adenocarcinoma, classified as pT2 pN1b (stage IIIA) according to UICC 8^th^ Edition, with preserved mismatch repair (pMMR) proteins. Due to the family history of colorectal and other cancers, the patient underwent genetic counseling and germline testing for hereditary cancer syndromes. No pathogenic mutations were detected. The initial carcinoembryonic antigen (CEA) level was 2.5 µg/L. Following surgery, adjuvant chemotherapy with capecitabine and oxaliplatin began in May 2023 and was completed in August 2023.

**Figure 1:**
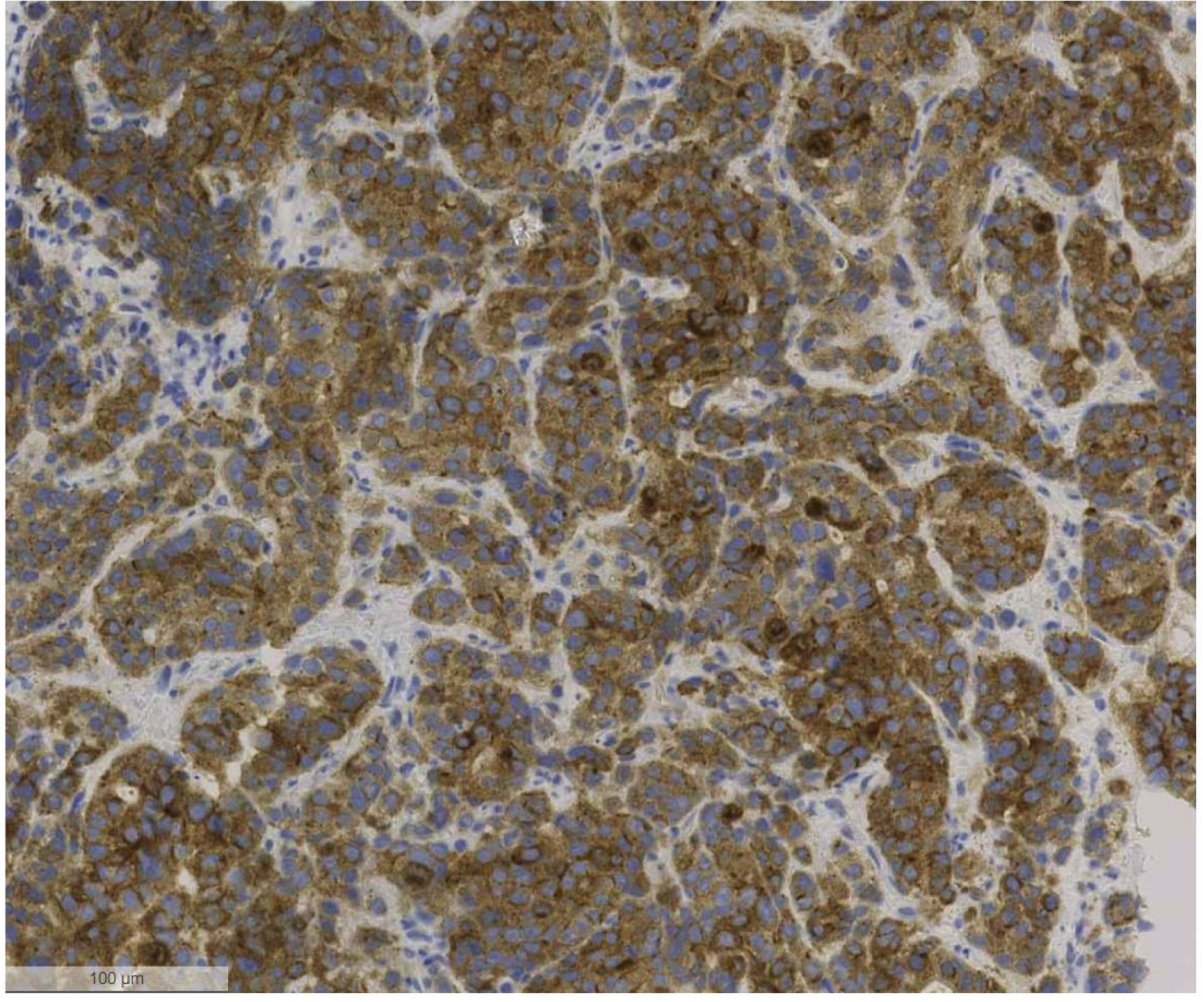
Positive ALK immunohistochemical staining of liver biopsy at 100 μm magnification.

In September 2023, the patient began experiencing fever and complained of upper right abdominal pain. A computed tomography (CT) confirmed multiple liver metastases, which were subsequently confirmed through a liver biopsy. Molecular testing using the FoundationOne^®^ CDx test (FOUNDATION MEDICINE, INC. CAMBRIDGE, MA, USA) revealed a number of ALK mutations, along with an *ALK-CEP44* fusion. Alongside IHC showed ALK expression, correlating with a low-level amplification of the p-arm of chromosome 2, locus of *ALK* gene (see **Figure 1**).

Despite the complex genotype which raised the question whether ALK was activated, first-line palliative therapy with FOLFIRI and panitumumab (as bevacizumab was contraindicated after a cholecystectomy which was performed few days ago) was initiated as this is one of the approved therapeutic options in this setting. However, given the circumstances and in anticipation of potential disease progression, a cost coverage request for alectinib was already submitted. The initial therapy led to a partial remission by December 2023, with symptom improvement and decreased tumor burden evidenced by laboratory and imaging findings.

**Figure 2:**
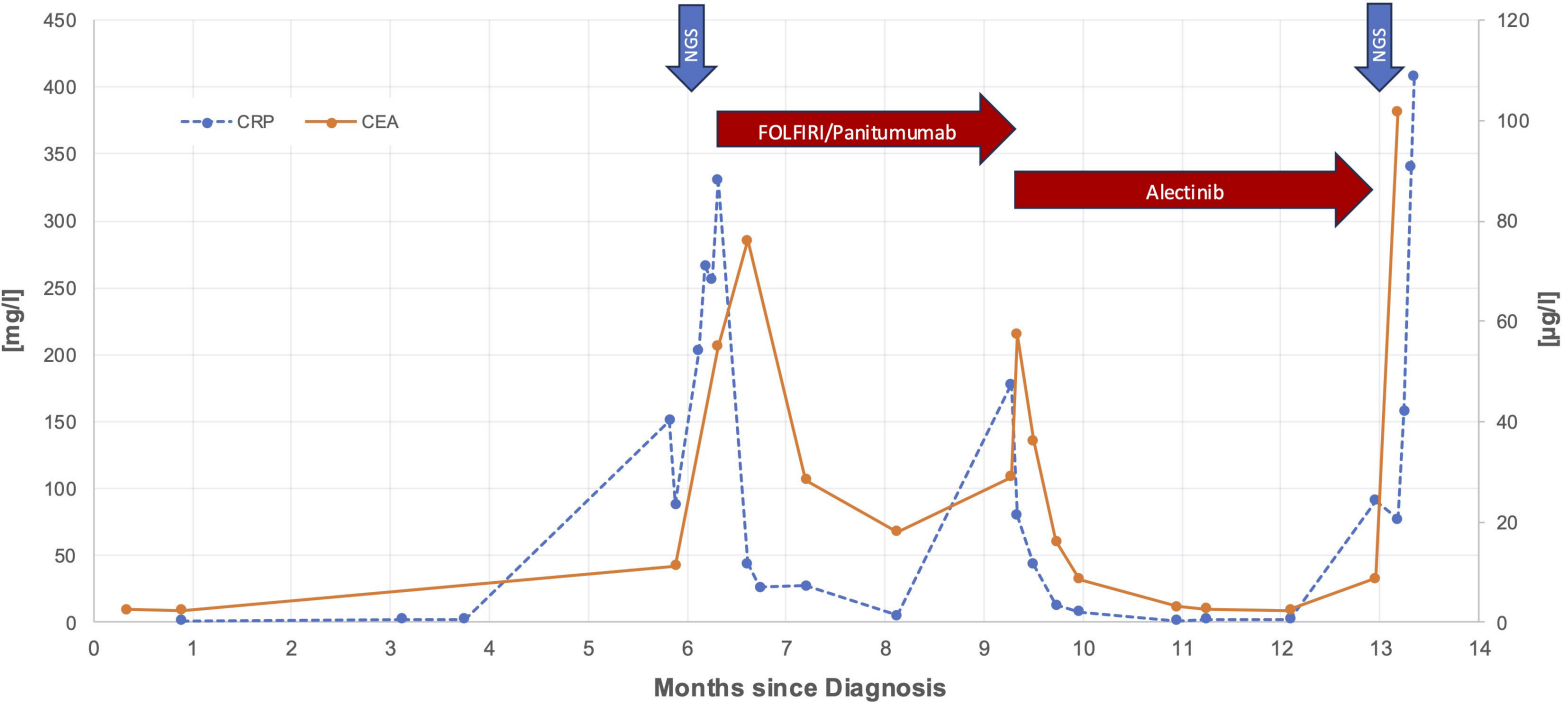
Time course of CEA and CRP levels during treatment period. Red arrows are highlighting responses to FOLFIRI/panitumumab and alectinib and blue arrows mark the time points of the first molecular analysis from liver biopsy and the second molecular analysis from peripheral blood, respectively.

Soon after, in January 2024, however, the patient experienced worsening fatigue, fever, jaundice and right upper quadrant pain. Blood tests indicated a significant increase in liver enzymes and the CEA rose sharply to 57.3 µg/L (see **Figure 2**). Imaging showed disease progression, which was the reason to switch therapy to the *ALK* inhibitor alectinib. The patient responded well to alectinib. Within a few days jaundice and fever subsided, abdominal pain disappeared, and CEA dropped to a normal value after one month. ECOG Performance state was 0. In March 2024, a CT scan showed a partial remission according to RECIST v1.1. Alectinib was continued.

**Figure 3:**
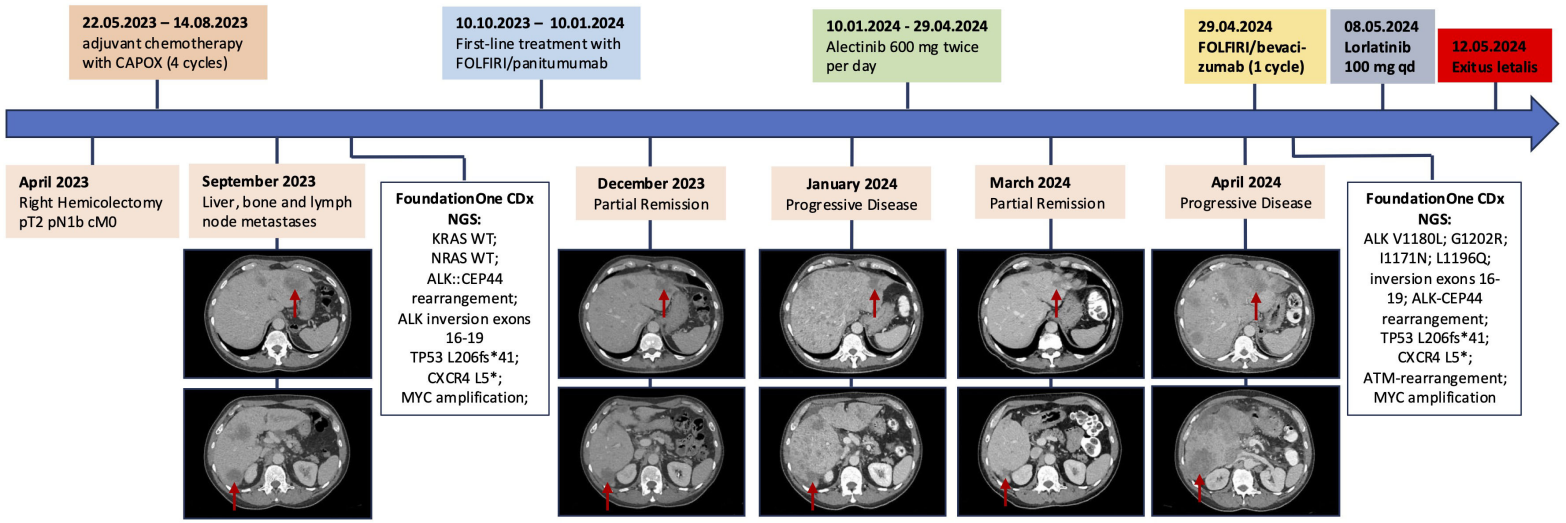
Timeline of the patient’s clinical history, summarizing therapeutic interventions, computed tomography findings with focus on liver metastases, and molecular profiling results at two time points. The red arrows each point to the liver metastases in segment II and segment VI, respectively.

However, only a few weeks later, in April 2024, again the patient’s condition swiftly deteriorated, with fever, severe fatigue, abdominal distention and now ascites. CEA and CRP increased again, and liver enzymes were markedly elevated. A CT scan confirmed extensive progression of liver metastases. A second NGS from liquid biopsy showed several new *ALK* mutations (see **Figure 3**). Considering the worsening liver function and aggressive disease spread, an emergency switch to FOLFIRI and bevacizumab was made as a bridging therapy while awaiting initiation of lorlatinib, a third generation *ALK*-inhibitor.

Treatment with lorlatinib was initiated shortly thereafter. Nevertheless, the patient’s liver function continued to decline, and his general condition worsened. Ultimately, the patient died in May 2024.

## Discussion

This case highlights a unique clinical scenario involving a mCRC with ALK overexpression by IHC and harboring multiple ALK mutations as well as an *ALK-CEP44* fusion. The patient responded quickly and well to the ALK inhibitor alectinib, but a swift emergence of multiple resistance-associated mutations and ultimately clinical resistance were observed. To our knowledge, the *ALK-CEP44* fusion has only been described once in a patient with NET and is unprecedented in colorectal cancer (10). *ALK* fusions in CRC are rare and most commonly involving fusion partners such as EML4, STRN and CAD (6, 7, 11). Typically, oncogenic *ALK* fusions result in constitutive kinase activation due to the loss of regulatory N-terminal domains, leading to sustained *ALK* phosphorylation and downstream oncogenic signaling (12). However, in this case, structural analysis suggested that the *ALK-CEP44* fusion lacked a fully intact kinase domain, likely preventing constitutive phosphorylation and downstream oncogenic signaling. Despite presumed absence of *ALK*-driven oncogenic activity, IHC demonstrated strong ALK expression. This correlated with a low-level amplification of the p-arm of chromosome 2, locus of *ALK* gene. Targeted therapy with alectinib led to a significant clinical, biochemical, and radiological response raising questions about the mechanism of action, potential off-target effects or alternative sensitivity mechanisms. However, the response was transient, underlining the potential limitations of monotherapy in cases with complex or incompletely understood molecular drivers.

The aggressive progression and drug resistance observed during the course of the disease are to some extent consistent with findings from other *ALK*-positive CRC cases. Studies on *ALK*-positive CRCs with fusion partners such as STRN and EML4 report similar rapid disease progression and limited efficacy of standard *ALK* inhibitors, often due to secondary mutations or activation of bypass signaling pathways (4, 7, 13). However, in this case, the aggressive progression and relatively short duration of response to alectinib may be attributed, at least in part, to the likely modest *ALK*-oncogenic activity of ALK expression.

CEP44 plays a critical role in centrosome cohesion, which is essential for maintaining chromosomal stability during cell division. By stabilizing rootletin, a key component of the centrosome linker, CEP44 ensures the structural integrity necessary for centriole-to-centrosome conversion (14, 15). Therefore, disruptions in CEP44 can lead to chromosomal instability, which may have further contributed to the aggressive progression observed in this case (15, 16).

After alectinib therapy four concurrent mutations in the *ALK* gene - V1180L, I1171N, G1202R and L1196Q - were identified. All of them have been associated with resistance to alectinib and other second-line *ALK*-inhibitors (17–19). The emergence of four distinct *ALK* resistance mutations following a short course of alectinib therapy is striking. In other studies, the presence of multiple *ALK* resistance mutations has typically been associated with prior exposure to two or more *ALK* inhibitors, suggesting a cumulative selective pressure over time (13). Additionally, it has been proposed that highly potent *ALK* inhibitors, such as lorlatinib, may selectively drive the development of multiple concurrent resistance mutations (20). In this case, the rapid acquisition of such mutations under the selective pressure of alectinib alone points to a high degree of tumor adaptability, likely contributing to the aggressive disease progression observed.

Among the identified mutations, L1196Q stands out due to its rarity and limited characterization in the literature. Unlike other *ALK* resistance mutations, L1196Q has been reported in only a few studies (18, 21). It has been shown to reduce the efficacy of lorlatinib by altering binding interactions, though its impact may be less pronounced compared to the structurally similar L1196M mutation, which is a known driver of lorlatinib resistance, especially in compound with G1202R (21, 22). Despite uncertainties about its efficacy in the context of the given mutations, lorlatinib was selected as the next therapeutic line. Unfortunately, the rapid progression of the disease precluded a meaningful assessment of therapeutic response. This highlights the limited targeted options after resistance and the urgent need for earlier access to next-generation inhibitors in this setting.

## Conclusion

This case underscores the importance of early testing for actionable driver mutations and timely planning of targeted therapies in aggressive *ALK*-positive colon cancers. Early access to such treatments could reduce delays and potentially prevent rapid progression, as observed here. Evidence from *ALK*-positive CRC cases suggests that initiating targeted therapy earlier, rather than adhering to standard second-line regimens, may benefit patients with high-risk *ALK* fusions and merits further investigation in clinical studies (7, 9). However, this case foremost highlights the challenges of predicting therapy response from molecular testing results. While some genetic aberrations confer a clear sensitivity to targeted therapy, most others do not. In particular, the interplay of complex genetic aberrations and the predictive potential of novel testing modalities, for example spatial transcriptomics and spatial proteomics, remain to be explored.

## Data Availability

The original contributions presented in the study are included in the article/**Supplementary Material**. Genetic testing was conducted via a commercial platform (FoundationOne® CDx), and raw data are not publicly available. Further inquiries can be directed to the corresponding author.
