# Supplementary material for: Case Report: Metastatic colorectal cancer with ALK–CEP44 fusion and rapid resistance development
Source: Front Oncol. 2025 Jun 18;15:1613235. doi: 10.3389/fonc.2025.1613235 (PMC12213891; doi:10.3389/fonc.2025.1613235)
Supplement: Supplementary file 1 [file DataSheet1.pdf]

Supplementary Information

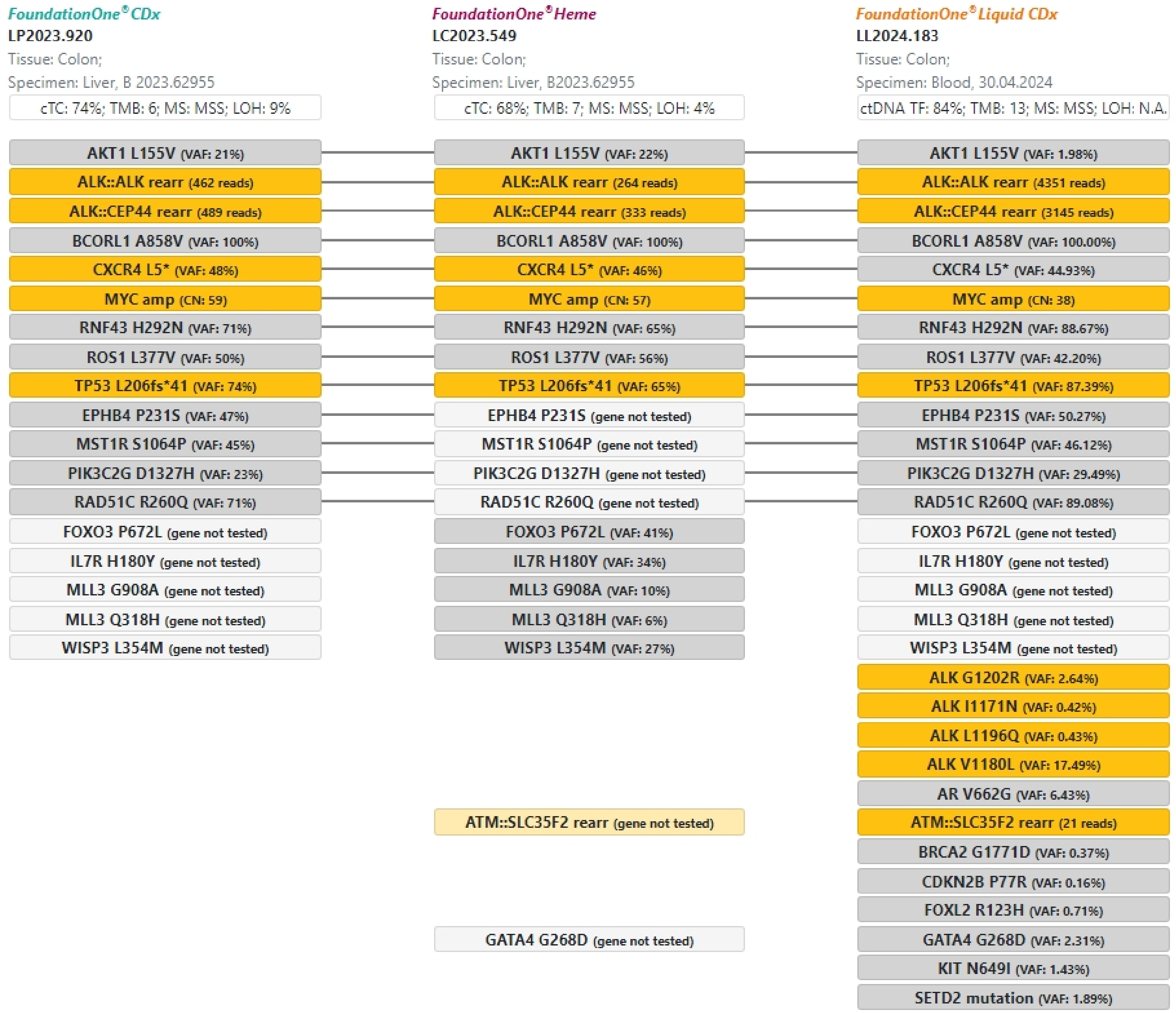

Figure S1: Comparison of the mutational tumor profile of the two conducted NGS using FoundationOne CDx.

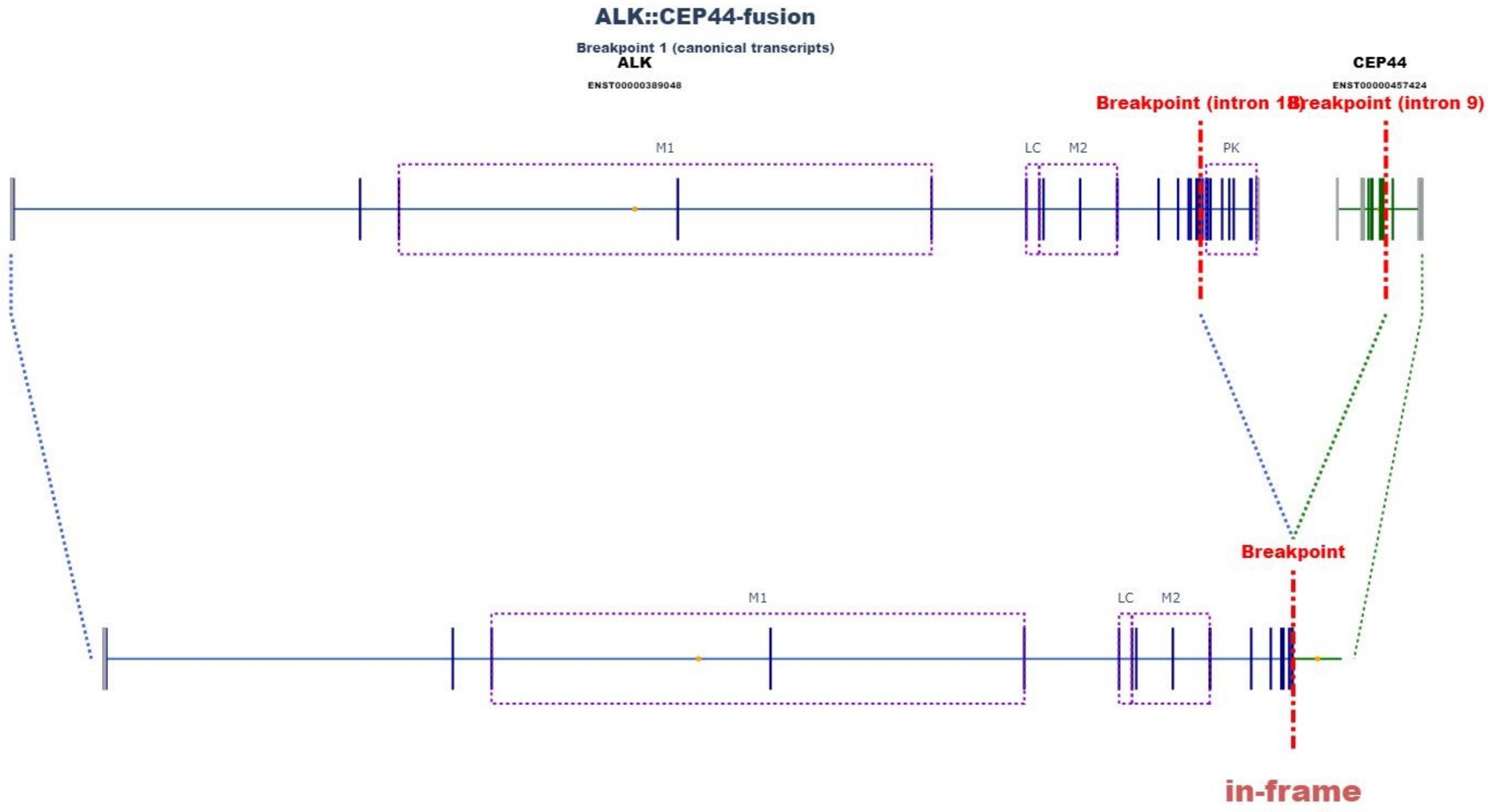

Figure S2: Visualisation of the ALK::CEP44 fusion.
