# Supplementary material for: Case Report: Metastatic colorectal cancer with ALK–CEP44 fusion and rapid resistance development
Source: Front Oncol. 2025 Jun 18;15:1613235. doi: 10.3389/fonc.2025.1613235 (PMC12213891; doi:10.3389/fonc.2025.1613235)
Supplement: Supplementary file 2 [file DataSheet2.pdf]

CARE case report guidelines

CARE Checklist of information to include when writing a case report

(cc) BY-NC-ND

Topic	Item	Checklist item description	Reported on Line
Title	1	The diagnosis or intervention of primary focus followed by the words "case report"	check
Key Words	2	2 to 5 key words that identify diagnoses or interventions in this case report, including "case report"	check
Abstract (no references)	3a	Introduction: What is unique about this case and what does it add to the scientific literature?	check
	3b	Main symptoms and/or important clinical findings	check
	3c	The main diagnoses, therapeutic interventions, and outcomes	check
	3d	Conclusion—What is the main "take-away" lesson(s) from this case?	check
Introduction	4	One or two paragraphs summarizing why this case is unique (**may include references**)	check
Patient Information	5a	De-identified patient specific information.	check
	5b	Primary concerns and symptoms of the patient.	check
	5c	Medical, family, and psycho-social history including relevant genetic information	check
	5d	Relevant past interventions with outcomes	check
Clinical Findings	6	Describe significant physical examination (PE) and important clinical findings.	check
Timeline	7	Historical and current information from this episode of care organized as a timeline	check
Diagnostic Assessment	8a	Diagnostic testing (such as PE, laboratory testing, imaging, surveys).	check
	8b	Diagnostic challenges (such as access to testing, financial, or cultural)	check
	8c	Diagnosis (including other diagnoses considered)	check
	8d	Prognosis (such as staging in oncology) where applicable	check
Therapeutic Intervention	9a	Types of therapeutic intervention (such as pharmacologic, surgical, preventive, self-care)	check
	9b	Administration of therapeutic intervention (such as dosage, strength, duration)	check
	9c	Changes in therapeutic intervention (with rationale)	check
Follow-up and Outcomes	10a	Clinician and patient-assessed outcomes (if available)	check
	10b	Important follow-up diagnostic and other test results	check
	10c	Intervention adherence and tolerability (How was this assessed?)	check
	10d	Adverse and unanticipated events	check
Discussion	11a	A scientific discussion of the strengths AND limitations associated with this case report	check
	11b	Discussion of the relevant medical literature **with references**.	check
	11c	The scientific rationale for any conclusions (including assessment of possible causes)	check
	11d	The primary "take-away" lessons of this case report (without references) in a one paragraph conclusion	check
Patient Perspective	12	The patient should share their perspective in one to two paragraphs on the treatment(s) they received	not available
Informed Consent	13	Did the patient give informed consent? Please provide if requested	Yes ☒ No ☐
